# Be Cognative: Cognates in the Rehabilitation of Cochlear Implant Users with German as a Second Language – A Computer‐Based Experiment

**DOI:** 10.1111/1460-6984.70138

**Published:** 2025-10-08

**Authors:** Susann Thyson, Maika Werminghaus, Thomas Klenzner

**Affiliations:** ^1^ Cochlear Implant Center, Department of Otorhinolaryngology Medical Faculty and University Hospital Düsseldorf, Heinrich‐Heine‐University Düsseldorf Germany

**Keywords:** aftercare, cochlear‐implantation, hearing disorders, multilingualism, rehabilitation

## Abstract

**Background:**

The rehabilitation of people with cochlear implants (PwCI) who speak more than one language poses significant challenges to healthcare systems, particularly in countries experiencing global migration. This study investigates the potential of cognates (CO) to enhance speech and language therapy for PwCI with German as a second language. A historically underserved group in rehabilitation contexts, speech and language therapy for PwCI speaking German as a second language is often affected by language barriers.

**Aim:**

This study aimed to investigate whether PwCI with bi‐ or multilingual backgrounds show an increased positive selection rate and a reduced latency in understanding auditorily presented CO compared to Non‐cognates (NCO), to identify a potential speech and language therapy approach for PwCI in the context of CI rehabilitation. In addition, the study investigated a possible correlation between the level of proficiency in the second language of PwCI and the frequency at which the second language is used in daily life with the selection of CO.

**Method:**

A computer‐based experiment was conducted using the open‐source software PsychoPy. The experiment involved 48 adult multilingual PwCI undergoing outpatient CI rehabilitation. Using a crossover design, participants completed auditory tasks involving CO and NCO at single‐word and sentence levels.

**Results:**

The study involved 48 multilingual PwCI with an average age of 55.7 years who received cochlear implants 66 months previously, on average. Participants spoke languages including Polish, Russian and Turkish, reflecting the linguistic diversity within the German population. The PwCI showed better performance in selecting and processing CO compared to NCO at both the single‐word and sentence levels, with significantly faster response times for CO. Daily use of German did not significantly affect CO selection speed at the single‐word level, but those who used German more often performed better with CO in sentences.

**Discussion and Conclusion:**

The results suggest that CO are processed faster and more accurately than NCO by multilingual PwCI. This finding highlights the potential of CO in auditory training and speech and language therapy, which is consistent with the existing literature on normal‐hearing individuals. CO can enhance comprehension exercises, which are crucial for speech and language therapy, and address the language barriers faced by multilingual PwCI and speech and language therapists. By incorporating exercises focused on CO into therapy, language and comprehension skills essential for multilingual PwCI could be enhanced, potentially improving their hearing abilities.

**WHAT THIS PAPER ADDS:**

*What is already known on this subject*

- Prior to our study, it was known that cognate‐based interventions in speech therapy for people with normal hearing have shown positive results. However, there has been limited research on the use of cognates in cochlear implant rehabilitation. This study aims to fill existing knowledge gaps and provide new insights that are crucial for optimising hearing rehabilitation strategies in cochlear implant recipients with a multilingual background.

*What this paper adds to the existing knowledge*

- As a result of this study, we received indications that cognate‐focused exercises can improve word and sentence processing in cochlear implant recipients with multilingual backgrounds. These findings highlight the potential of cognates to improve speech and language therapy outcomes. They open up new opportunities for optimising rehabilitation strategies in this population. These findings could lead to tailored interventions that improve comprehension skills for multilingual cochlear implant users.

*What are the potential or actual clinical implications for this work?*

- The study has clear clinical implications. Integrating cognates into speech and language therapy for multilingual people with cochlear implants may improve their auditory training outcomes. This approach may improve their language processing and communication skills, potentially leading to more effective and personalised rehabilitation strategies.

## 1 Introduction

Adequate management of multilingual, hearing‐impaired patients presents a major challenge in the context of global migration (Arici et al. 2019; Buqammaz et al. 2021; Rabinowitz et al. 2005). Some countries, such as Germany and the United Kingdom, face the challenge of integrating individuals with multilingual and often diverse cultural backgrounds into the existing health care system (Gerlinger 2022; Mead and Roland 2009). To date, the data on the use of preventive and (re)habilitative health services by people with a migration background is incomplete. There is a lack of scientific surveys and contributions that allow a reliable overall assessment of the care situation across all healthcare sectors (Sheikh et al. 2004). In the context of the cochlear implant (CI) provision process, patients with a migration background and German as a second language in Germany receive care through outpatient, semi‐inpatient, or inpatient rehabilitation measures (Dazert et al. 2020). The language barrier and the perceived low effectiveness of therapy provided by monolingual speech and language therapists are significant hurdles in the treatment process (Ellahham 2021; Hahn et al. 2020; Kritikos 2003; Timmins 2002). Communicative limitations are particularly evident during diagnostics, assessment and counselling interviews, and speech and language therapy (Slade and Sergent 2023; Taylor and Jones 2014).

**FIGURE 1:**
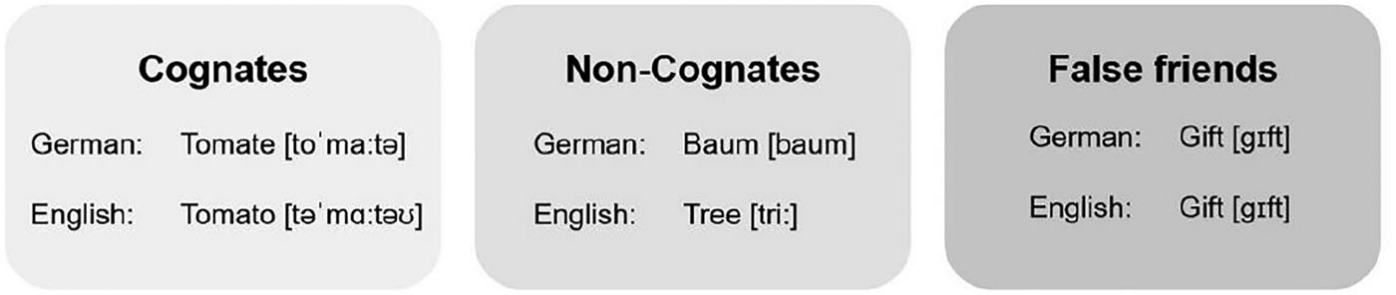
Definition of cognates.

The majority of the world's population is bi‐ or multilingual and uses more than one language in their daily lives. There are over six thousand languages spoken in the world, belonging to different language families. Within these language families exist grammatical and lexical relationships (Möller 2011). Words with shared meanings and close phonological similarity can be found across different languages. These words are referred to as cognates (CO) or related words (see Figure 1, definition of cognates) (Costa et al. 2005; Dijkstra 2009; Möller 2011). Non‐cognates (NCO) are words that do not have phonological and semantic similarities across different languages. Words that share phonological similarity in two or more languages but do not have the same meaning are called false friends or false CO (Al‐Wahy 2009; Simpson Baird et al. 2016).

Studies have found evidence indicating faster and more accurate receptive processing of CO compared to NCO in multilingual individuals without hearing impairments (Costa et al. 2005; Groot et al. 2002; Lemhöfer et al. 2004). Within speech and language therapy for adult patients, CO are already successfully utilised in aphasia therapy settings (Ansaldo and Saidi 2014; Grasso et al. 2021). CO facilitate the transfer of therapy effects from the treated to the untreated language, particularly in semantic therapy approaches, thereby enhancing health care efficiency (Ansaldo and Saidi 2014).

Rehabilitation for adult patients with cochlear implants (PwCI) focuses on improving and restoring speech understanding, enhancing social participation and facilitating vocational reintegration (Dazert et al. 2020). Speech and language therapy after cochlear implantation includes auditory identification and discrimination exercises at the level of sounds, phonemes, words, sentences and text. It also includes exercises in directional hearing, pragmatics and prosody perception (Illg 2017). Speech and language therapy for PwCI speaking German as a second language is often affected by language barriers. Supporting speech and language therapists in CI rehabilitation by using CO could be an approach in the treatment of PwCI with German as a second language. After an extensive literature review, we did not find any approach to the treatment of bi‐ or multilingual adult PwCI in the context of CI rehabilitation. Therefore, the aim of this study was to investigate whether PwCI with bi‐ or multilingual backgrounds show an increased positive selection rate and a reduced latency in understanding auditorily presented CO compared to NCO, in order to identify a potential speech and language therapy approach for PwCI in the context of CI rehabilitation. In addition, this study investigated a potential correlation between the selection of CO, the level of proficiency in the second language of PwCI and the frequency of its use in daily life.

## 2 Methods

This study is registered in the German Registry of Clinical Trials under number DRKS00030907. The study was reviewed by the Ethics Committee of Heinrich Heine University Düsseldorf and received a positive ethics vote under application no. 2023/2308_01. The study complied with the ethical principles of medical research in humans (as outlined in the Declaration of Helsinki).

### 2.1 Participants

The study included adult PwCI with German as a second or third language. At the time of testing, these PwCI were undergoing outpatient CI rehabilitations and met the following inclusion criteria: they had been fitted with at least one CI, spoke German as a second or third language, had received their initial CI fitting at least 2 months previously, had acquired their hearing loss later in life and had no exposure to sign language at home or through formal education and had no diagnosed cognitive impairment at the time of testing. Prior to the start of the experiment, all participants were assessed for language background and proficiency in German using the Common European Framework of Reference for Languages (CEFR) (Council of Europe 2024). Demographic data and additional information regarding language background were collected using a custom‐designed questionnaire. Additionally, data on tone and speech audiometry were gathered from all PwCI.

In accordance with the national German AWMF Guideline on CI Care (Söver et al. 2023), all participants received at least a basic course of post‐implantation therapy, which included structured speech‐language and pedagogical intervention as part of their cochlear implant rehabilitation.

### 2.2 Material and Procedure

A computer‐based experiment was conducted using the open‐source software PsychoPy (Peirce 2007). German words and sentences, consisting of CO and NCO, were recorded by a qualified native speaker of German with experience in the recording of auditory stimuli. The recording took place in a soundproof booth using a RØDE NT‐USB+ microphone. The recordings were stored on a PC, and the stimuli were subsequently edited using Audacity software to ensure a consistent output volume for all recorded words and sentences.

**FIGURE 2:**
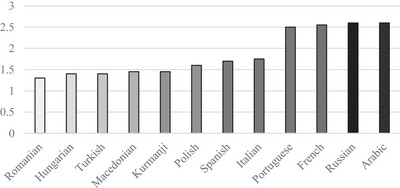
Levenshtein distance. The ordinate axis shows the calculated Levenshtein distance. The abscissa axis shows the 12 languages of the PwCI.

Twenty CO and 20 NCO were selected. The CO were extracted from databases and dictionaries and cross‐referenced across 12 languages. All lexical items were nouns with syllable lengths ranging from one to five and were selected based on a minimum frequency value of 2 in the DWDS Frequency Barometer; each item occurred at least five times in the DWDS Core Corpus to ensure linguistic accessibility for participants. Twenty NCO, unique phonologically and semantically to German, served as control stimuli. The phonological proximity of the CO was determined using the Levenshtein distance (Levenshtein 1966). The CO were transcribed and compared using the International Phonetic Alphabet. Figure 2 shows the phonological distance between the German CO and its counterpart in one of the other 12 languages. On average, the selected CO in Portuguese (M = 2.50 | SD = 1.50), French (M = 2.55 | SD = 1.40), Russian (M = 2.60 | SD = 1.70) and Arabic (M = 2.60 | SD = 2.50) show the largest phonological distance between the CO compared to the German CO. The CO in Romanian (M = 1.30 | SD = 1.10), Hungarian (M = 1.40 | SD = 0.90), Turkish (M = 1.40 | SD = 1.30) and Macedonian (M = 1.45 | SD = 1.40) show the smallest phonological distances between the CO.

The experiment consisted of two parts. One section contained 40 words (20 CO and 20 NCO), while the other section contained 40 sentences (20 with CO and 20 without CO as objects). To prevent one part of the experiment from influencing the other, the study was conducted using a crossover design.

**FIGURE 3:**
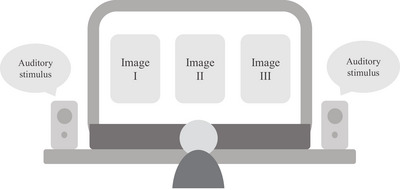
Experiment procedure.

The experiment was conducted on a 15‐in. laptop in a quiet room. The audio reproduction of the target items was delivered through commercially available desktop speakers connected to a laptop. As shown in Figure 3, three images were presented alongside each auditorily presented word or phrase. One picture showed the target item that corresponded to the auditorily presented word or phrase, while another showed a phonological distractor that sounded phonologically similar to the target item. A third picture showed a semantic distractor that had a strong semantic similarity to the target item.

Before the start of the study, the image material was checked for accuracy of representation: all images were presented visually to independent, normal‐hearing adults (*n* = 48) in a pilot study. Participants rated the correspondence between the images and the words used in the study using an online survey tool on a sliding scale from 0 to 10. The image material consisted of freely available online images and those for which official licences had been obtained for use. Overall, the images received an average rating of 9.28 (SD = 0.76), indicating high accuracy in representing the presented word and confirming strong correspondence.

### 2.3 CEFR

To provide a detailed description of the sample and allow for correlation analyses, PwCI with an MB completed a self‐assessment of their German language skills using the CEFR. The CEFR is an internationally established framework for describing and assessing language proficiency across various skill areas. It differentiates six levels of proficiency: A1, A2, B1, B2, C1 and C2, ranging from basic to highly proficient users, and offers a structured approach to evaluating language competence across a broad spectrum. The framework allows for self‐assessment in listening, reading, spoken interaction, spoken production, and writing. When needed, the CEFR was made available in participants' first languages. Participants assigned one of the six proficiency levels to each language domain, and a mean score was calculated based on these self‐ratings.

## 3 Results

### 3.1 Participants

A total of 26 female and 22 male PwCI with a multilingual background participated in the study. At the time of testing, the PwCI had an average age of 55.7 years (SD = 16.6) and had been implanted with a CI on average 66 months (SD = 66.9) previously. Hearing loss was assessed as the pure‐tone average (decibel hearing level) over four octave frequencies (0.5, 1, 2 and 4 kHz) (PTA (dB HL)). Word recognition in silence was separately evaluated for both ears at 65 dB SPL and 80 dB SPL using the Freiburg monosyllabic test. As shown in Table 1, the PwCI had a PTA (dB HL) of 33.07 (SD = 7.59) in the CI‐implanted ear and a PTA (dB HL) of 58.69 (SD = 29.5) in the unaided contralateral ear (participants without hearing loss in the contralateral ear were not included). Evaluation of the Freiburg monosyllabic word recognition test in silence for the CI‐implanted ear (*n* = 48) showed an average speech understanding of 46.91% at 65 dB SPL and 57.55% at 80 dB SPL. For the separately measured unaided contralateral ear, the results were 51.50% at 65 dB SPL (*n* = 10) and 59.33% at 80 dB SPL (*n* = 15), respectively.

**TABLE 1:** Hearing‐related patient informations.

Subjects (*n*)	48	
**Sex ♀ | ♂ (%)**	54 | 46	
**Age at testing (years) (M|SD)**	55.70 (16.60)	
**Time since cochlear implantation (CI) (month) (M|SD)**	66 (66.90)	
**CI‐System (%)**		
Cochlear	64.59	
MED‐EL	27.08	
Advanced Bionics	6.25	
Oticon	2.08	
**Supply Status (%)**		
bilateral (CI)	6.25	
bimodal (CI + Hearing Aid)	62.50	
Single‐sided deafness	16.67	
unilateral (CI + unaided on the opposite ear)	14.58	
**WHO grades of hearing loss (unaided opposite ear) (%)**		
Unilateral	16.67	
Mild or moderate hearing loss	14.58	
Moderately severe or severe hearing loss	31.25	
Profound, complete or total hearing loss	37.50	
**Audiometric data**	**CI**	**Unaided opposite ear**
PTA (500 Hz, 1000 Hz, 2000 Hz, 4000 Hz) (M|SD)	(*n* = 48) 33.07 (7.59)	(*n* = 45)^b^ 58.69 (29.50)
Speech comprehension[Link], c 65 dB (M|SD)	(*n* = 48) 46.91 (25.68)	(*n* = 10^)^ d 51.5 (37.05)
Speech comprehension[Link], c 80 dB (M|SD)	(*n* = 48) 57.55 (26.45)	(*n* = 15^)^ d 59.33 (30.17)

^a^Measured with Freiburger monosyllabic speech test.

^b^ Calculation without the three bilaterally CI‐supplied subjects.

^c^ In bilaterally supplied subjects, the last implanted CI was measured.

^d^ For some subjects, monosyllable comprehension could not be determined due to the degree of hearing loss or missing data.

All participants were residents in Germany at the time of the study and had lived in Germany for an average of 23.26 years (SD = 9.99). Due to a change in their living environment, they were bi‐ or multilingual and learned German informally in their daily lives. Table 2 shows the language distribution of the participants. All participants were literate and reported an average of 9.31 years of schooling (SD = 2.52). All participants included in the study were postlingually deaf, having acquired hearing loss after the development of spoken language.

**TABLE 2:** Language Profile of the participants.

Subjects	*n* = 48	*n* = 48	*n* = 10
Language	L1 (%)	L2 (%)	L3 (%)
Polish	35.42		
Russian	14.58	2.08	20
Italian	10.42	2.08	
Turkish	12.5		
Kurmanji	6.25		
Romanian	4.17		
Hungarian	4.17		
Portuguese	4.17		
Spanish	2.08		20
Macedonian	2.08	2.08	
Arabic	2.08	2.08	
French	2.08		
German		87.5	60

*Note*: L1 = Language 1 (first language); L2 = Language 2 (second language); L3 = Language 3 (third language).

### 3.2 Experiment‐Related Results

Two tests were employed to assess the normality of distribution: the Kolmogorov–Smirnov test and the Shapiro–Wilk test. For both CO and NCO at the single‐word and sentence levels, the null hypothesis of normal distribution of data was assumed. However, the results of the Shapiro–Wilk test and the Kolmogorov–Smirnov test (corrected according to Lilliefors) were significant (CO at the single‐word level: *p* < 0.001, NCO at the single‐word level: *p* < 0.001, CO in sentences: *p* < 0.001, NCO in sentences: *p* < 0.001), indicating that the data did not follow a normal distribution, and thus, the null hypothesis was rejected.

In total, the experiment included *n* = 1920 tasks for CO (960 single‐word CO and 960 CO in sentences) and *n* = 1920 tasks for NCO (960 single‐word NCO and 960 NCO in sentences) completed by PwCI. There were no missing values. PwCI incorrectly selected CO as the target item in 76 tasks, which corresponds to 3.96% of the total CO tasks. Specifically, 37 tasks (48.68%) were single‐word CO, and 39 tasks (51.32%) were CO in sentences. For NCO, there were 322 incorrectly chosen tasks, accounting for 16.77% of all NCO tasks. Of these, 139 tasks (43.17%) were single‐word NCO, and 182 tasks (56.52%) were NCO in sentences.

Overall, CO were selected correctly more frequently than NCO across all experiments. Among incorrectly answered tasks by PwCI, there was a minimal difference between selecting semantic distractors (49.52%) and phonological distractors (50.48%). To assess the latency of selecting CO and NCO, the non‐parametric Wilcoxon signed‐rank test for paired samples was conducted using IBM SPSS Statistics (for Macintosh) v29.0.1. The effect size was calculated using the Pearson correlation coefficient (r) for statistically significant results. The results indicate that, at the single‐word level, CO (Mdn = 0.618) were recognised significantly faster than NCO (Mdn = 1.268); asymptotic Wilcoxon test: *z* = −14.088, *p* < 0.001, *n* = 960. The effect size was *r* = −0.455, which, according to Cohen (1992), corresponds to a medium‐sized effect. Similarly, in the sentence context, sentences containing CO (Mdn = 0.643) were recognised significantly faster than sentences without CO (Mdn = 1.233); asymptotic Wilcoxon test: *z* = −11.293, *p* < 0.001, *n* = 960. The effect size was *r* = −0.364, indicating a medium‐sized effect according to Cohen's guidelines (1992).

During the experiment, spontaneous participant remarks were documented by the research team. Of the 48 participants, comments were recorded for 26 individuals with cochlear implants (PwCI). These remarks could be categorised into three thematic areas: (1) relevance to speech audiometry, (2) perceived benefit in rehabilitation and (3) personal or emotional connection. Specifically, 26.92% of participants provided unsolicited feedback suggesting that the use of CO could also be beneficial in speech audiometric testing, noting that they perceived such stimuli as easier or more intuitive to recognise. 19.23% indicated that they believed CO had been helpful during therapeutic auditory training as part of their CI rehabilitation. Another 19.23% made spontaneous comments reflecting a personal connection to the CO, highlighting links to their first language, feeling acknowledged in their multilingual identity, or recalling past experiences related to multilingualism.

### 3.3 Language‐Related results

**FIGURE 4:**
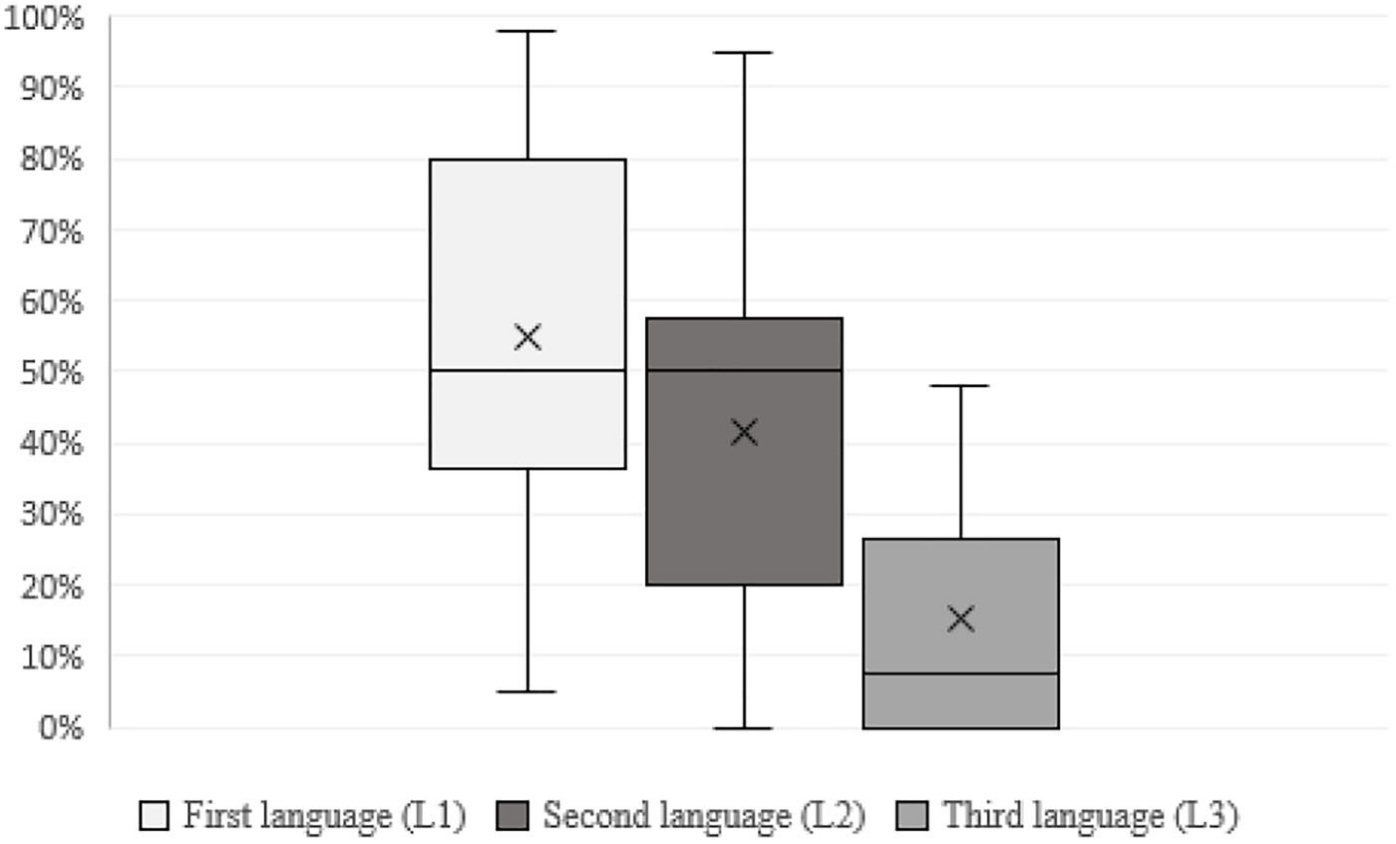
Subjective use of language in daily life. Values were determined by subject self‐assessment. The data refer to a retrospective period of 1 month.

In the study, 33 participants reported using their first language ≥ 50% in daily life, while 26 participants whose second language was German reported using German ≥ 50% in daily life (Figure 4). It was hypothesised that PwCI with a multilingual background who use German ≥ 50% in daily life would select CO faster than NCO at the single‐word level. To test this hypothesis, the Mann‐Whitney U test was conducted. The results indicate that PwCI in our study group using German ≥ 50% in daily life (Mdn = 0.584) did not select CO faster at the single‐word level than those using German < 50% in daily life (Mdn = 0.667), exact Mann–Whitney *U* test: *U* = 110049.000, *p* = 0.309. The effect size, according to Cohen (1992), was *r* = −0.03, which corresponds to a negligible effect. Therefore, the hypothesis was not supported. Additionally, it was hypothesised that PwCI with a multilingual background who use German ≥ 50% in daily life would select CO in sentences faster than NCO. The results show that PwCI using German ≥ 50% in daily life (Mdn = 0.593) did select CO in sentences faster than those using German < 50% in daily life (Mdn = 0.685), exact Mann‐Whitney *U* test: *U* = 103184.000, *p* < 0.009. The effect size was *r* = −0.08, indicating a negligible effect size according to Cohen's guidelines (1992).

**FIGURE 5:**
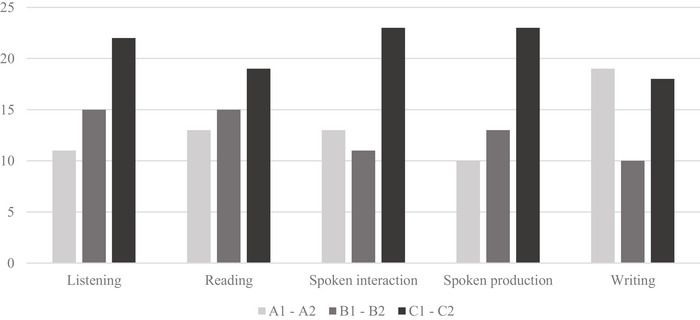
Language proficiency in German. The ordinate axis shows the number of subjects who indicated the level of proficiency in this category. The Abscissa axis shows the communicative abilities. Subjects assessed language proficiency using the Common European Framework of Reference for Languages: Learning, Teaching, Assessment (CEFR). The CEFR was provided to the subjects in their first language or in the language in which they felt most confident. The CEFR organises language proficiency at six levels: A1–A2 (basic user), B1–B2 (independent user) and C1–C2 (proficient user).

Figure 5 shows the PwCIs' self‐assessed language background and proficiency in German using the CEFR. On average, the PwCIs rated their German language skills as independent (B1/B2) or competent (C1/C2) (M = 2.12/SD = 0.24). The PwCIs rated their German language skills best in the category of *listening* (M = 2.23/SD = 0.80) and thus most often as independent or competent, followed by *reading* (M = 2.08/SD = 0.86), *writing* (M = 1.94/SD = 0.92), *spoken production* (M = 2.19/SD = 0.90) and *spoken interaction* (M = 2.17/SD = 0.90).

A Kruskal–Wallis *H* test was conducted to examine whether reaction time for CO differed significantly across the three language proficiency levels: A1/A2, B1/B2 and C1/C2. For this analysis, a global CEFR level was derived from patients’ self‐reported proficiency in German by calculating a mean score, which was then used to assign each patient to an overall language proficiency level: A1/A2, B1/B2 and C1/C2. The test assessed whether differences in language proficiency were associated with variations in CO selection speed. The mean ranks of language proficiency levels were 21.22 for A1/A2, 26.04 for B1/B2 and 24.16 for C1/C2. The results indicated that there was no statistically significant difference in reaction times for CO among the three language proficiency levels, *H*(2) = 0.988, *p* = 0.610. These findings suggest that language proficiency does not significantly impact reaction time in CO selection. A Kruskal–Wallis H test was also conducted to examine whether reaction time for CO in sentences differed significantly across the three language proficiency levels: A1/A2, B1/B2 and C1/C2. The mean ranks of language proficiency levels were 23.94 for A1/A2, 22.75 for B1/B2 and 23.56 for C1/C2. The results indicated that there was no statistically significant difference in reaction times for CO in sentences among the three language proficiency levels, *H*(2) = 0.058, *p* = 0.972. These findings suggest that language proficiency does not significantly impact reaction time in CO selection.

In a Kendall's tau b correlation analysis investigating the relationship between reaction time for CO and time of exposure in the second language German, no statistically significant correlation was observed, *τ*_β_ = −0.086, *p* = 0.452. This result suggests that, within the sample studied, the amount of time individuals have been exposed to German as a second language does not significantly influence their reaction time for the task. For the reaction time of CO in sentences and the time of exposure to the second language German, no statistically significant correlation was found using Kendall's tau‐b analysis, *τ*_β_ = −0.133, *p* = 0.244.

A Spearman's Rho correlation analysis was conducted to examine the relationship between reaction time for CO and duration of CI use. The results revealed no statistically significant correlation between these variables *rho*(48) = 0.170, *p* = 0.248. This result suggests that the duration of CI use in multilingual PwCI does not have a statistically significant effect on reaction time in the processing of CO. A Spearman's Rho correlation analysis was also conducted to examine the relationship between reaction time for CO in sentences and duration of CI use. The results revealed a statistically significant positive correlation between these variables *rho*(48) = 0.330, *p* = 0.022. In contrast, this result indicates that CO in sentences are related to the duration of CI use.

A Spearman's Rho correlation analysis was also conducted to examine the potential relationship between hearing loss (measured by PTA4) and reaction time during the selection of CO and CO in sentences. The analysis revealed no statistically significant correlation for either condition (CO: *rho*(48) = −0.092, *p* = 0.534; CO in sentences: *rho*(48) = 0.119, *p* = 0.419. These findings suggest that, within the present sample, hearing loss may not have a measurable impact on reaction time for CO and CO in sentences, and therefore may not substantially influence the comprehension of these items in this specific context.

## 4 Discussion

The analysis of the experiment revealed significant results indicating faster selection of CO compared to NCO. Additionally, CO were more frequently chosen correctly. These findings apply to both CO at the single‐word level and within sentences. Similar outcomes have been reported in the literature concerning individuals with normal hearing ability (Costa et al. 2000). The faster and more frequent correct understanding of CO compared to NCO suggests a meaningful applicability of CO within CI rehabilitation. These results confirm that the use of CO could be beneficial in speech and language therapy (Ansaldo and Saidi 2014). Word and sentence comprehension exercises, such as word identification or differentiation, are essential components of speech and language therapy in CI rehabilitation. They also provide the basis for further training in areas such as text comprehension, pragmatics and prosody (Illg 2017). Using CO in speech and language therapy interventions could make listening and auditory training more accessible to PwCI with a multilingual background. CO could serve as a communication bridge within speech and language therapy and thus alleviate the perceived problems resulting from the language barrier and the feeling of low effectiveness of therapeutic interventions (Kritikos 2003). In our study, participant comments also pointed in this direction, as several individuals provided positive feedback regarding the usefulness of CO and their potential for broader application. Moreover, the results showed that neither the participants' level of German language proficiency nor their length of residence in Germany had a significant impact on reaction time during correct CO selection. This suggests that CO may serve as a valuable resource in rehabilitation, even for patients with only basic proficiency in the therapy language or who have only recently begun acquiring that language.

However, the findings of our study relate exclusively to the receptive processing of CO, whereas the majority of existing studies focus on expressive language abilities. Some research has shown that individuals with high proficiency in their second language tend to produce a greater number of CO in expressive tasks (Blumenfeld et al. 2016; van Hell and Tanner 2012).

We also examined whether a better hearing threshold, as measured by PTA4, was associated with faster reaction times during correct CO selection. For our group of PwCI, no such correlation was found. This finding suggests that CO could be applicable in CI rehabilitation settings, as they appear to be processed effectively even by individuals with poorer hearing thresholds and multilingual backgrounds. However, the duration of CI use did appear to influence the correct selection of CO within sentence contexts. As our experimental CO stimuli consisted solely of nouns rather than verbs, this may reflect the previously observed effect whereby sentence‐level comprehension of CO improves when nouns are used (Bultena et al. 2014).

PwCI with multilingual backgrounds appear to be intersectionally affected by their hearing impairment, as they face not only challenges associated with impaired hearing but those associated with reduced language access as well, particularly within the context of CI rehabilitation. In auditory diagnostics and speech and language therapy, it becomes especially evident that PwCI with multilingual backgrounds are at a disadvantage due to their bi‐ or multilingualism (Suite et al. 2023). To address this disadvantage, the use of CO can provide support in the therapeutic setting. It can also strengthen the therapist‐patient relationship, as the use of CO can help communicate the therapist's commitment and effort to the PwCI. Multilingual PwCI in particular often feel misunderstood and taken less seriously in the clinical treatment setting (Mead and Roland 2009; Sheikh et al. 2004). Similarly, monolingual speech and language therapists can use CO to address their personal perceptions of uncertainty and the barriers impeding the therapeutic effectiveness (Kritikos 2003).

The majority of respondents' first languages were Polish, Russian and Turkish, in line with the average distribution of languages spoken in Germany besides German (Statistisches Bundesamt (DESTATIS) 2023). PwCI who assessed their language skills using the CEFR rated themselves as competent, particularly in the area of listening. This may be related to the fact that when learning a new language, receptive vocabulary is usually more extensive than expressive vocabulary (Oh and Mancilla‐Martinez 2021). In order to utilise these resources in speech and language therapy, the experiment focused on assessing the receptive performance of PwCI with multilingual backgrounds. In addition, patient self‐assessment of their own second language competency can help therapists identify current levels of language proficiency, set individualised therapy goals and assess their patients' needs. Patients' self‐assessment of their second‐language writing skills as low should also be taken into account in speech and language therapy; writing exercises are often an additional component of therapy in CI rehabilitation. To ensure optimal speech and language therapy, digital translation tools or AI‐based systems could be used to improve communication skills and support therapy with specifically generated exercises.

### 4.1 Limitations

It should be noted that a small proportion of participants (14.58%) exhibited mild to moderate residual hearing in the unaided contralateral ear. While this subgroup may experience auditory input differently from participants with bilateral profound hearing loss, we chose not to exclude them from the analysis. These individuals are nonetheless part of the population of PwCI and continue to face substantial hearing‐related challenges. We argue that overall functional hearing ability should be considered when characterising participants. However, we acknowledge that residual hearing may have had a subtle influence on individual performance and encourage future research to explore such effects more systematically.

Another limitation of the present study concerns the variability in participants' exposure to and acquisition of the German language. The participants acquired German informally through daily interactions and lived in Germany for several years prior to cochlear implantation, often with limited or distorted auditory access. While this reflects real‐world conditions for many PwCI, it introduces heterogeneity in phonological development and language experience. Additionally, detailed data on formal language instruction and pre‐implantation speech and language therapy could not be collected, as this information was unavailable in medical records.

## 5 Conclusion

The results of this study suggest that CO could be utilised effectively in auditory training and speech therapy rehabilitation. Exercises could be integrated into the areas of word and sentence comprehension, which serve as foundational skills for more complex exercises in text comprehension, pragmatics and prosody. Since there are currently no specific therapy approaches for PwCI with a multilingual background, CO could present a meaningful addition to speech and language therapy.

Therapeutic approaches should consider individual language habits and abilities to optimise auditory and speech therapy. This could provide multilingual PwCI with better access to auditory exercises and training, ultimately enhancing their communication skills and quality of life. Understanding the emotional and social impact of improved language processing through the use of CO could aid the development of more holistic therapy approaches that not only enhance linguistic abilities but also foster confidence in therapy among multilingual PwCI.

Future research could build on and improve the insights gained in this study by considering, for instance, the long‐term effects of CO on therapy outcomes within speech and language therapy. Additionally, the effects of differences in multilingualism between individuals should be investigated further. Different linguistic backgrounds and specific language pairings may have varying impacts on the efficacy of auditory training and rehabilitation. Furthermore, developing and evaluating specialised digital tools and apps could provide a pathway for utilising CO as an entry point to speech and language therapy. Investigating the effects of CO in various acoustic environments could also be meaningful in this context.

## Ethics Statement

The study has received a positive ethical approval from the Ethics Committee of the Heinrich Heine University Düsseldorf, application number 2023–2308_1. It adheres to the ethical principles for medical research involving human subjects (Declaration of Helsinki).

## Conflicts of Interest

This study was conducted as part of the first author‘s DrPH (Public Health) doctorate at the Medical Faculty of Heinrich‐Heine‐University (HHU) and University Hospital Düsseldorf. All authors declare no financial or non‐financial competing interests.

## Data Availability

The data used in this study are available on request from the author via e‐mail.
